# Acetaldehyde production by *Rothia mucilaginosa* isolates from patients with oral leukoplakia

**DOI:** 10.1080/20002297.2020.1743066

**Published:** 2020-03-21

**Authors:** Abdrazak Amer, Aine Whelan, Nezar N. Al-Hebshi, Claire M. Healy, Gary P. Moran

**Affiliations:** a Division of Oral Biosciences, School of Dental Science, Trinity College Dublin, Dublin Dental University Hospital, Dublin, Ireland; b Department of Genetic Engineering, Biotechnology Research Center (BTRC), Tripoli, Libya; c School of Chemical and Pharmaceutical Sciences, Technological University, Dublin, Ireland; d Oral Microbiome Research Laboratory, Maurice H. Kornberg School of Dentistry, Temple University, Philadelphia, PA, USA; e Division of Oral and Maxillofacial Surgery, Oral Medicine and Oral Pathology, School of Dental Science, Trinity College Dublin, Dublin Dental University Hospital, Dublin, Ireland

**Keywords:** Bacteria, *Rothia*, metagenome, alcohol, acetaldehyde, oral leukoplakia

## Abstract

*Rothia mucilaginosa* has been found at high abundance on oral leukoplakia (OLK). The ability of clinical isolates to produce acetaldehyde (ACH) from ethanol has not been investigated. The objective of the current study was to determine the capacity of *R. mucilaginosa* isolates recovered from OLK to generate ACH. Analysis of *R. mucilaginosa* genomes (n = 70) shows that this species does not normally encode acetaldehyde dehydrogenase (ALDH) required for detoxification of ACH. The predicted OLK metagenome also exhibited reduced ALDH coding capacity. We analysed ACH production in 8 isolates of *R. mucilaginosa* and showed that this species is capable of generating ACH in the presence of ethanol. The levels of ACH produced (mean = 53 µM) were comparable to those produced by *Neisseria mucosa* and *Candida albicans* in parallel assays. These levels were demonstrated to induce oxidative stress in cultured oral keratinocytes. This study shows that *R. mucilaginosa* can generate ACH from ethanol *in vitro* at levels which can induce oxidative stress. This organism likely contributes to oral ACH levels following alcohol consumption and the significance of the increased abundance of *R. mucilaginosa* in patients with potentially malignant disorders requires further investigation.

## Introduction

Recent studies have linked changes in the oral microbiome with the development of oral cancers, including oral squamous cell carcinoma (OSCC) [1,2]. Many hypotheses have been generated to explain how changes in the oral microbiome could trigger or exacerbate carcinogenesis [3,4]. Microbial production of acetaldehyde (ACH) from ethanol has been proposed as one such mechanism by which bacteria could induce mutations in oral keratinocytes [5–7]. Although alcohol consumption is a known risk factor for OSCC, ethanol itself is not carcinogenic. However, when alcohol enters the human body, it can be reduced by host or microbial encoded alcohol dehydrogenases (ADH) to produce ACH [7]. ACH has been classified as a carcinogenic substance by the International Agency for Research on Cancer (IARC) [8]. It has been shown to have tumorigenic activity in animal models and exposure of cultured cells to ACH *in vitro* results in the generation of DNA adducts and chromosomal abnormalities [5,9,10]. Studies have shown that the oral microbiome plays a role in generating ACH in the oral cavity following alcohol consumption [5,11]. *In vitro* studies have shown that incubation of saliva with ethanol results in the generation of ACH and that saliva from smokers, alcohol drinkers and those with poor oral hygiene generate higher levels of ACH *in vitro* [11–13]. Patients with OSCC and oral lichenoid disease have also been shown to harbor acetaldehyde generating microorganisms at lesional sites, and that cultures from smokers generated greater levels of acetaldehyde compared to non-smokers [14].

ACH produced by microorganisms can be further reduced to acetate by acetaldehyde dehydrogenases (ALDH). In many bacteria, this ALDH activity is encoded by the *adhE* gene, encoding a bifunctional alcohol and aldehyde dehydrogenase. This enzyme has been shown to be required for fermentative growth, efficient use of sugar alcohols and may play a role in ethanol tolerance in bacteria [15–17]. Fermentative bacteria that do not express ALDH activity have been shown to generate high levels of ACH as they do not have the capacity to detoxify ACH to acetate [18,19]. To date, most studies that have analysed the capacity of common oral microbes to generate ACH in the presence of ethanol have implicated *Neisseria* spp. and *Candida albicans* as the most potent producers [18,20–22]. ACH production by streptococci is strain specific and is associated with a minority of strains that exhibit reduced levels of enzymatic ALDH activity required to convert ACH to acetate [19,23].

Recently, healthy individuals with a high capacity to generate ACH in their saliva were found to harbour a high proportion of *Rothia mucilaginosa* in the salivary microbiome [24]. *R. mucilaginosa* is a Gram-positive, aerobic, non-spore forming bacterium belonging to the phylum Actinobacteria [25–27]. *R. mucilaginosa* is a member of the healthy oral microbial community and microbiome studies suggest that this species accounts for up to 6% of bacteria found on the tongue [28,29]. Recently, *R. mucilaginosa* levels have been shown to be affected by dietary nitrate levels and this bacterium has been implicated in nitrate reduction in the oral cavity [30].

Interestingly, our previous analysis of the oral microbiome in patients with OLK showed that *R. mucilaginosa* was more abundant on OLK compared to healthy tissue [29]. We have previously hypothesised that this increase in *R. mucilaginosa* levels could potentially contribute to the malignant transformation of OLK [31]. In this study, we wished to investigate this further by determining the capacity of *R. mucilaginosa* isolates recovered from patients with OLK to generate ACH. If this species has a high capacity to generate ACH, OLK patients may be exposed to potentially carcinogenic levels of ACH that could play a role in the development of OLK and/or malignant transformation of OLK to OSCC.

## Material and methods

### In silico *analysis of oral bacteria genomes*

We examined the distribution of predicted ALDH encoding genes within the genomes of common oral bacteria using the PATRIC database (https://patricbrc.org) [32]. The PATRIC database is a large database of high quality, consistently annotated microbial genomes. We included 652 complete and fully annotated bacterial genome sequences representing the dominant genera of bacteria present in the oral microbiome. Genes predicted to encode enzymes with aldehyde dehydrogenase activity (EC 1.2.1.10) were included in our analysis.

### Metagenome prediction

PICRUSt can be used to predict gene family abundance (e.g. the metagenome) in microbial communities for which only marker gene (e.g. 16S rRNA gene) data are available [33]. This analysis was carried out on our previously published microbiome dataset comparing the microbiomes of oral leukoplakia (OLK) and normal contralateral (NC) healthy tissues from 36 patients [29]. We have previously shown that the OLK microbiome exhibits increased levels of *R. mucilaginosa* and reduced levels of streptococci compared to NC healthy mucosa from the same patient [29]. Normal contralateral (NC) sites were deemed healthy following examination by an experienced consultant in oral medicine and acted as an age and oral hygiene matched control to the diseased sites. The reads were reclassified with Mothur using Wang’s method and Greengenes 97% clustered OTUs (version 13.5) as reference [34]. The reads were then assigned to OTUs based on their taxonomy (phylotype command) and the generated file was converted into a BIOM (Biological Observation Matrix) table. Output files showing the distribution of genes (catalogued by KEGG ontologies) and pathway abundances were generated. These files were analysed using the linear discriminant analysis (LDA) effect size (LEfSe) method to identify categories enriched in OLK or healthy NC tissues [35].

### *Isolation of* Rothia mucilaginosa

Mucosal swabs recovered from OLK patients were cultured on *Rothia* selective Brain Heart Infusion (BHI) agar plates. All samples were taken with informed consent and with approval of the Joint Hospital’s Research Ethics Committee, Dublin. Selective BHI agar media consisted of standard BHI agar supplemented by sodium selenite (Na_2_SeO_3_) at concentration of 50 mg/l and colistin at concentration 10 mg/l [26]. Bacterial DNA extraction was carried out as described [36] and PCR amplification of the 16S gene was carried out using the primers 27F and 1492R. Following Sanger DNA sequencing, species identification was carried out by BLAST analysis of the Human Oral Microbiome Database (HOMD) and each query resulted in the identification of *R. mucilaginosa* as the best match to the query sequences, with between 98% and 100% nucleic acid sequence identity (Table S1).

### Growth in ethanol

*R. mucilaginosa* isolates and several common oral microbes, including *Streptococcus mitis* NCTC12261, *Streptococcus gordonii* DL1, *Neisseria mucosa* DSM-17611 and *Candida albicans* 132A, were grown in the presence of ethanol. Strains were incubated in BHI broth with ethanol at a final concentration (v/v) of 0.0%, 2.0% (0.44 M) and 4.0% (0.87 M). Growth was measured by determining the OD600 nm at hourly intervals. Doubling times were determined from the exponential phase of the growth curve using Prism 8.3 (GraphPad Software).

### Aldehyde detection

Two methods of aldehyde detection were used. Firstly, a colorimetric aldehyde detection assay kit (Sigma-Aldrich, Co. Wicklow, Republic of Ireland) was used for total aldehyde quantification. Secondly, to verify these data and to specifically detect ACH, we also applied solid phase microextraction (SPME) with Gas chromatography-mass spectrometry (GC-MS) to indicated samples. We analysed 8 strains of *R. mucilaginosa* including the reference strain *R. mucilaginosa* DY-18 (DSM-20746). Several common oral microbes were included as controls, including *Streptococcus mitis* NCTC12261, *Streptococcus gordonii* DL1, *Neisseria mucosa* DSM-17611 and *Candida albicans* 132A.

Bacteria were grown in BHI broth at 37°C to late exponential phase (OD600 nm 0.8), harvested by centrifugation and washed three times in sterile PBS. A suspension measuring 0.5 at OD600 nm was prepared. A 450 µl volume of this suspension was transferred to a 2 ml volume plastic screw capped tube (Sarstedt, Germany) and 50 µl of ethanol solution was added to yield a final concentration of 10 or 100 mM. After two hours incubation, the reaction was frozen at −80°C. Total aldehydes were measured using a colorimetric aldehyde detection assay kit (Sigma-Aldrich) according to the manufacturer’s instructions. Samples were incubated with the detection reagent for 1 h and the coloured reaction product was measured by determining the absorbance at 540 nm in a 96-well plate reader (Genios, Tecan Group Ltd, Switzerland). ACH concentration in each sample was estimated from a standard curve using the positive control values from a serial dilution of a glutaraldehyde standard.

Gas-chromatography mass spectrometry GC-MS was carried out at the Mass Spectrometry laboratory at Technological University Dublin (TU Dublin) using a Varian 3800 gas chromatograph with a Varian Saturn 2000 GC/MS/MS detector. The standard acetaldehyde solutions (1 µM, 5.0 µM, 100 µM, and 200 µM) were extracted using a 65 µm PDMS/DVB coated SPME fibre (Sigma-Aldrich). Prior to each use, the fibre was blanked and exposed to the headspace of a PTFE-capped 4 ml glass vial containing 1 mL of a 10 mg/ml aqueous solution of O-2,2,4,5,6-(pentafluorobenzyl) hydroxylamine (PFBHA, 98%; Sigma-Aldrich). The fibre was then exposed to the ACH standard for 10 min. The PFBHA formed oxime adducts (cis- and trans- isomers) with the ACH. The fibre was then inserted into the injection port of the GC-MS and left to desorb for 2 min. For analysis of the bacterial samples, a blanked fibre was first exposed to PFBHA for 10 min, then the fibre was exposed to the headspace of the reaction samples for 10 min. The fibre was then desorbed in the injection port for 2 min and the analysis was left to run. Any ACH present in the samples then formed the same oxime adducts as previously mentioned.

### Measurement of ACH induced ROS production

In order to determine whether ACH (25–100 µM; corresponding to the levels produced by *R. mucilaginosa in vitro*) could induce stress in oral keratinocytes, we exposed cultured TR146 keratinocytes to ACH *in vitro*. Oral keratinocytes were cultured as described by Connolly *et al*. [37]. To measure reactive oxygen species (ROS) production, 2ʹ,7ʹDichlorodihydrofluorescein diacetate (DCF-DA; Sigma Aldrich) was used, which is non-fluorescent unless oxidized by intracellular ROS. TR146 cells were incubated with 100 µM DCF-DA in 96-well plates for 1 h at 37°C. Measurement of intracellular ROS was performed by incubating TR146 cells with either serum-free medium containing ACH (25, 50, 75, 100 µM) for 3 h with or without 1 h pretreatment with 100 µM of the antioxidant N-acetylcysteine (NAC). H_2_O_2_ (30 mM) was used as a positive control and PBS was added as a negative control. Cells were then washed two times in Hank’s balanced salt solution buffer (HBSS) and the fluorescence (485 nm excitation/530 nm emission) was measured using a Tecan plate-reader (Genios, Tecan). The effect of ACH at these levels was also examined using a fluorescence microscope (Zeiss Instruments). DCF-DA fluorescence was visualized with a GFP filter set (485 nm excitation/530 nm emission) and Hoechst stained nuclei visualized with a standard DAPI filter set.

## Results

### Acetaldehyde dehydrogenase genes in oral bacteria

**Figure 1.**
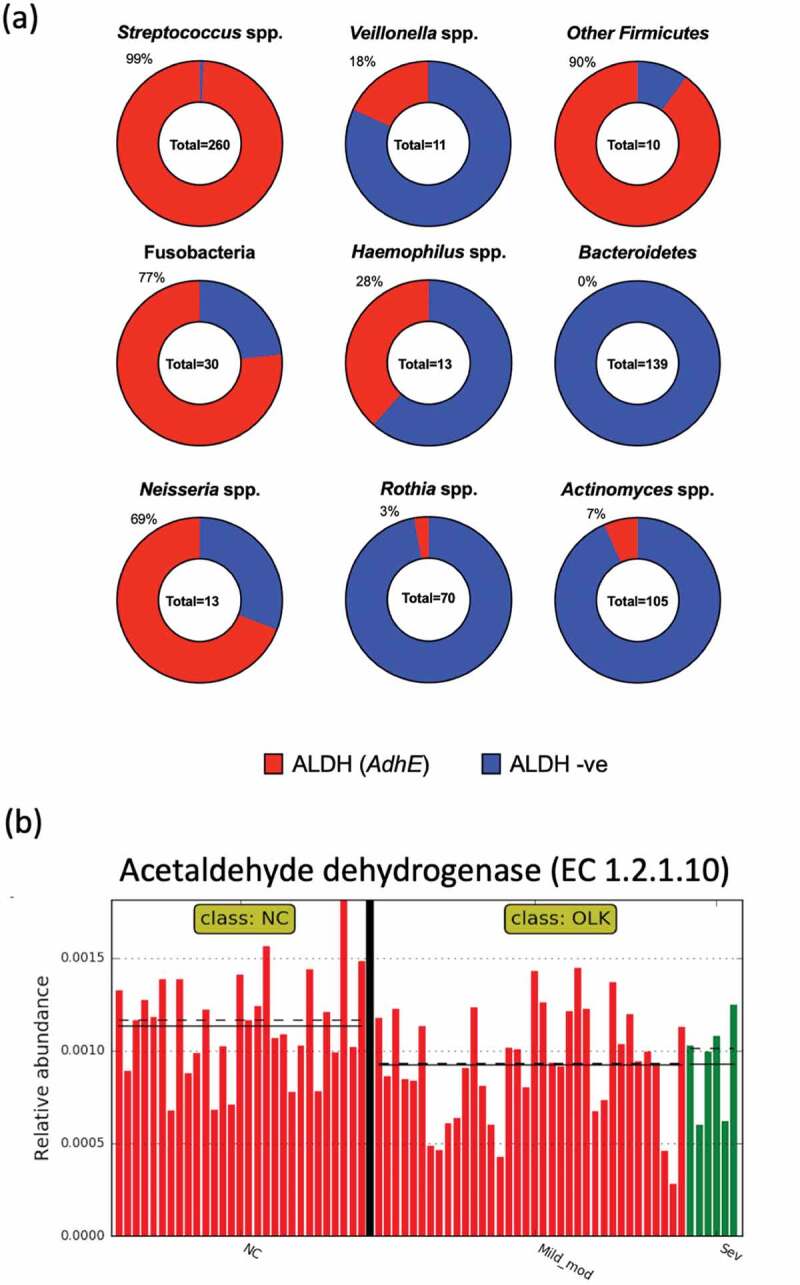
(a) Graphic representation of ALDH coding capacity in different taxonomic groups of oral bacteria, investigated using the PATRIC database. The percentage of strains with a predicted ALDH encoding gene is indicated and highlighted in red. Other Firmicutes includes *Granulicatella* spp. (n = 6) and *Gemella* spp. (n = 4). Fusobacteria includes *Fusobacterium* spp. (n = 15) and *Leptotrichia* spp. (n = 15). Bacteroidetes includes *Porphyromonas* spp. (n = 71), *Prevotella* spp, (n = 66) and *Tannerella* spp. (n = 3). (b) Plot generated in LEfSe showing the increased abundance of acetaldehyde dehydrogenase enzyme (EC 1.2.1.10) encoding genes in the metagenomes of samples from healthy NC mucosa compared to OLK (P = 0.008). OLK are categorized as in mild to moderate dysplasia (red) and severe dysplasia (green). Straight and dotted lines correspond to mean and median values, respectively

We examined the distribution of predicted ALDH encoding genes in 652 complete and fully annotated oral bacterial genome sequences (Figure 1(a)). A total of 260 streptococcal genomes were analysed and these were found to contain 479 putative ALDH encoding genes, with 99% of genomes harbouring at least 1 gene. Genomes of Bacteroidetes (*Porphyromonas* spp., *Tannerella* spp. and *Prevotella* spp.) did not contain any predicted ALDH genes. Other than Bacteroidetes, the Actinobacteria, including *Actinomyces* spp. and *Rothia* spp., had the lowest percentages of predicted ALDH encoding genes (7% and 3%, respectively; Figure 1(a)).

Of the 70 *Rothia* spp. genomes analysed, only two strains encoded genes with homology to ALDH encoding genes. *R. mucilaginosa* strain 204_RMUC harboured a gene encoding a protein with 100% identity to a protein in *S. oralis* strain Uo5 (*adhE*, PATRIC ID: fig|927666.3.peg.160). Secondly, *R. mucilaginosa* strain 382_RMUC contained a gene encoding a protein with 100% identity to an ALDH encoding gene from *Neisseria mucosa* ATCC 25996 (PATRIC ID: fig|546266.6.peg.2301). Alignment of these loci identified flanking regions of homology to *S. oralis* and *N. mucosa* suggesting that two strains acquired these genes during recent horizontal gene transfer events (Figure S1). BLAST analysis of both proteins from *R. mucilaginosa* strains 382_RMUC and strain 204_RMUC against the remaining 68 *Rothia* genomes did not identify any additional putative ALDH enzymes.

### Oral leukoplakia metagenome has reduced ALDH coding capacity

We next applied the metagenome prediction tool PICRUSt to our previously published 16S rRNA analysis of the microbiome of OLK and healthy NC mucosal samples from 36 patients [29]. Using LEfSe analysis (Figure S2), we observed increased levels of genes encoding acetaldehyde dehydrogenases (K04072) in NC healthy tissue compared to OLK samples (P = 0.008; Figure 1(b)). There was no significant difference between OLKs exhibiting different degrees of dysplasia. Examination of the pathway level data showed that OLK samples exhibited enrichment for genes involved in lipopolysaccharide biosynthesis compared to the NC samples (Figure S3). Carbohydrate metabolism was generally more prevalent in NC tissues (Figure S3).

### *Growth and ACH production by* R. mucilaginosa *in the presence of ethanol*

**Figure 2.**
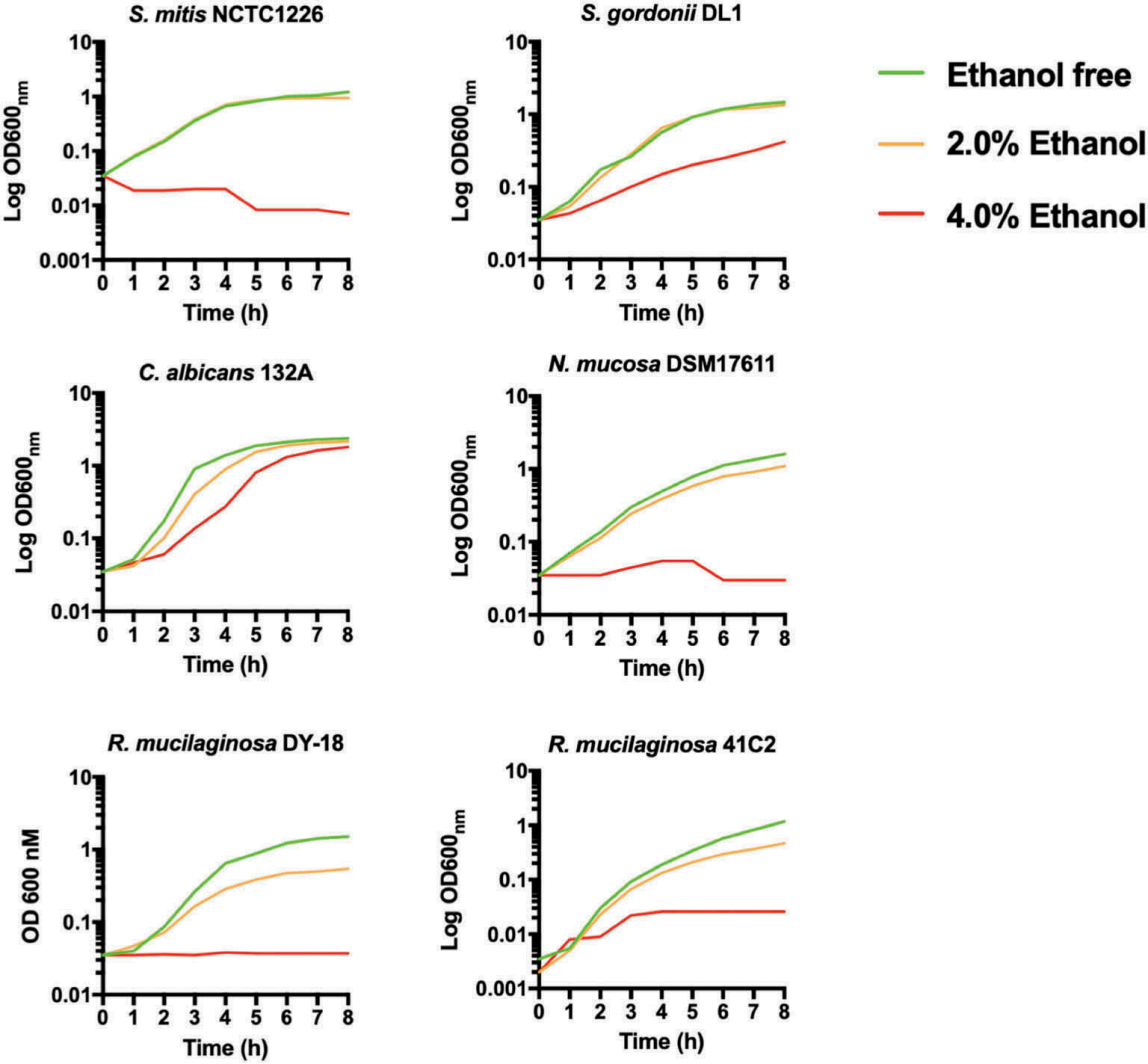
Growth of oral bacteria in BHI at 37°C in the presence of ethanol (0%, 2% and 4% v/v). Plots are the results of three separate replicate experiments

Next, we determined if lack of ALDH affected the susceptibility of *R. mucilaginosa* to high concentrations of ethanol in comparison to other common oral microbes (Figure 2). In general, all bacteria were severely inhibited by 4% ethanol, whereas *C. albicans* 132A was still capable of growth following a short lag period (Figure 2). At 2% ethanol, *R. mucilaginosa* and *Neisseria mucosa* exhibited reduced growth (Figure 2) and longer doubling times (Table S2) whereas *S. mitis* and *S. gordonii* were largely unaffected by growth in 2% ethanol (Figure 2; Table S2).

**Figure 3.**
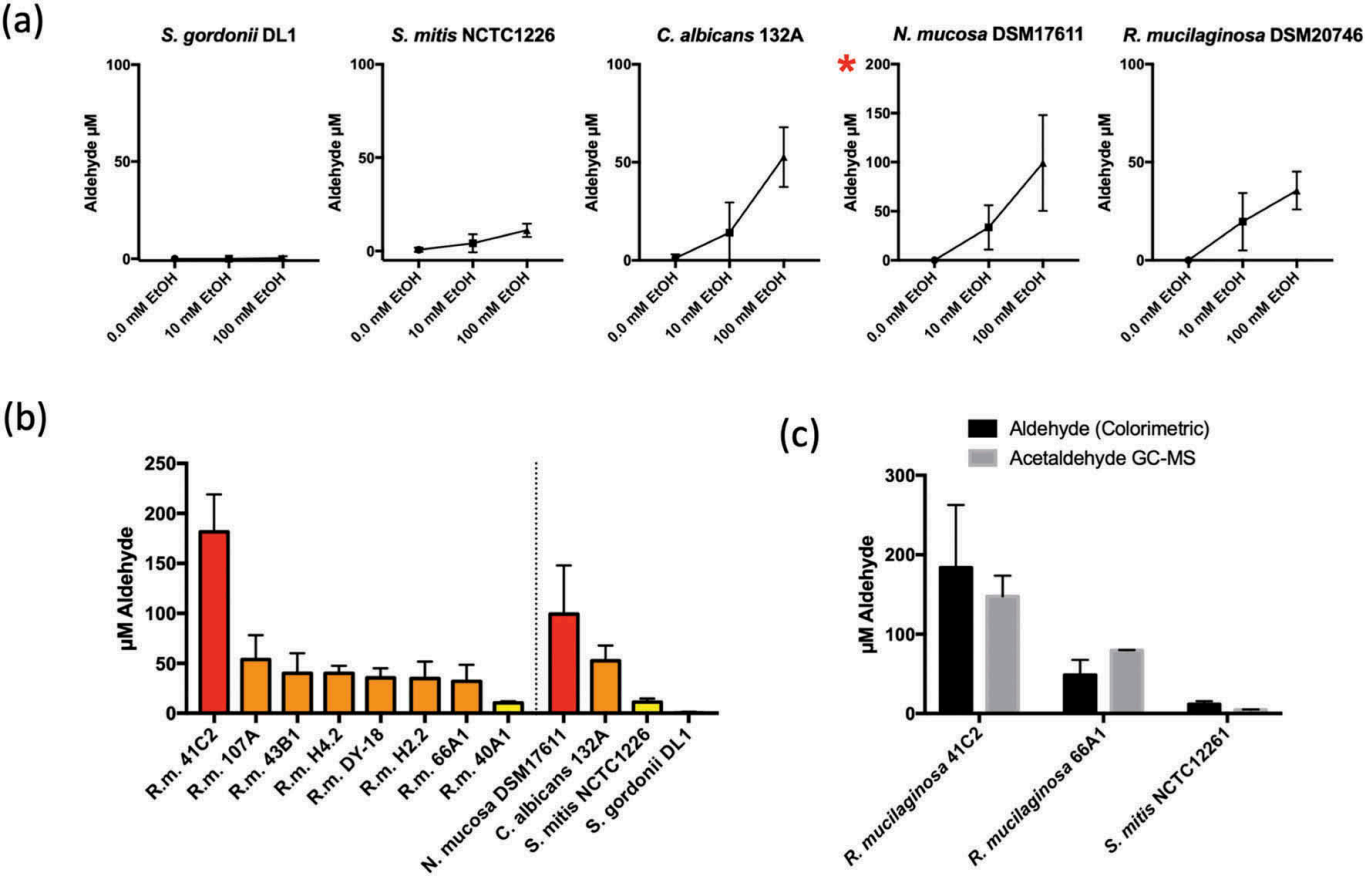
(a) Aldehyde production by representative oral microorganisms in the presence of 10 mM and 100 mM ethanol. Aldehyde was detected using a colorimetric assay. Data is the average of three separate experiments. Red asterisk indicates different axis scale. (b) Aldehyde production by clinical isolates of *R. mucilaginosa* in the presence of 100 mM ethanol. Red: >100 µM; Orange: 30–100 µM; yellow: < 20 µM. (c) Comparison of aldehyde and acetaldehyde measurements by colorimetric and GC-MS analysis following incubation of strains in 100 mM ethanol

Next, a colorimetric assay was carried out to determine the level of aldehydes produced by *R. mucilaginosa in vitro* in the absence and presence of subinhibitory concentrations ethanol (10 mM and 100 mM). Known ACH producing species *C. albicans* 132A and *N. mucosa* DSM17611 yielded on average approximately 53 µM and 100 µM aldehyde in the presence of 100 mM ethanol, respectively (Figure 3(a)). Aldehyde levels in *S. gordonii* DL1 were undetectable and *S. mitis* NCTC1226 yielded ~ 10 µM (Figure 3(a)). The type strain of *R. mucilaginosa*, DY18, yielded approximately 40 µM aldehyde under the same conditions (Figure 3(a)). Analysis of the 7 additional clinical isolates of *R. mucilaginosa* indicated an average yield of 53 µM aldehyde (Figure 3(b)). Most isolates (6/8) produced 40–60 µM with the exception of 40A1 which was consistently lower (10 µM) and 41C2, which consistently produced the highest levels of aldehyde observed here at approximately 180 µM (Figure 3(b)).

Solid phase microextraction (SPME) with Gas chromatography-mass spectrometry (GC-MS) was used to specifically measure ACH levels in additional experiments, confirming that the data from colorimetric measurements corresponded to ACH production. These analyses confirmed the production of approximately 150 µM and 80 µM ACH in strains *R. mucilaginosa* 41C2 and 66A9 respectively (Figure 3(c))

### Effect of ACH on oxidative stress levels in oral keratinocytes

**Figure 4.**
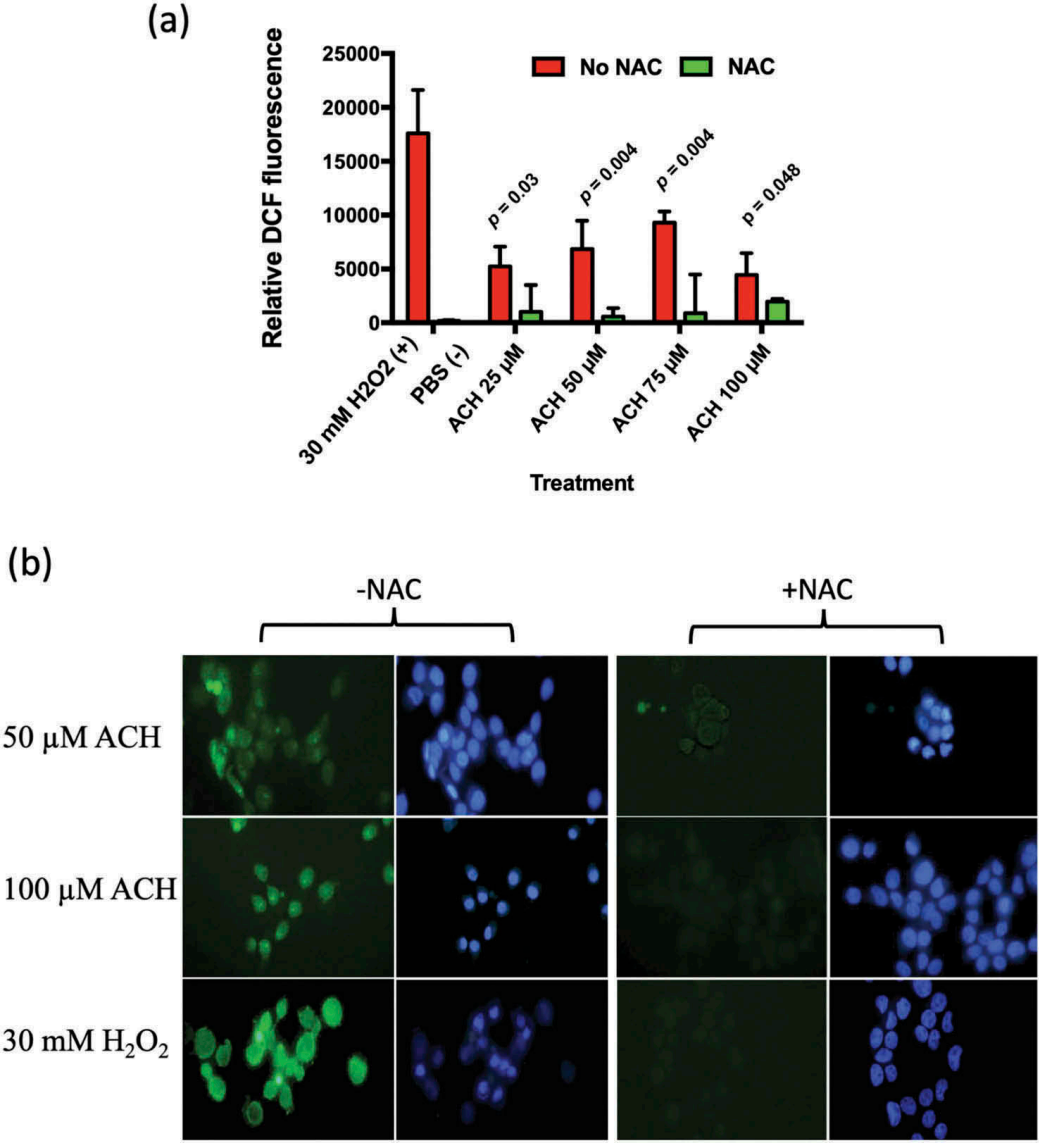
(a) Showing ACH induced oxidative stress in TR146 oral keratinocytes. Cells were incubated with ACH (25 µM – 100 µM) in 96 well plates. 30 mM H_2_O_2_ was used as positive control and medium containing PBS as a negative control. Reactive oxygen species (ROS) were detected by measuring DCF-DA fluorescence in a microplate reader (Genios, Tecan) in the presence and absence of N-acetylcysteine (NAC). Results are the average of three separate experiments. (b) Showing fluorescence in TR146 cells stained with DCF-DA to detect ROS (green) and Hoechst to detect nuclei (blue). Cells were preincubated in 100 µM of DCF-DA with or without NAC. Cells were then exposed to stress (50 or 100 µM ACH or 30 mM H_2_O_2_) and visualized using a Zeiss epifluorescence microscope

After measuring the level of ACH produced by *R. mucilaginosa* strains, we examined whether these levels were capable of inducing oxidative stress in TR146 oral keratinocytes. Increasing levels of ACH (25 to 75 µM) resulted in increased DCF-DA fluorescence (Figure 4(a)). The addition of the antioxidant NAC reduced the level of ROS induced fluorescence (Figure 4(a)). Interestingly, the highest concentration of ACH did not yield significantly higher levels of fluorescence, which may be due to cell toxicity or death.

The effect of ACH at these levels was also examined using fluorescence microscopy. The addition of ACH (50 or 100 µM) or H_2_O_2_ (30 mM) resulted in bright green fluorescence, which could be reduced by the addition of NAC (Figure 4(b)).

## Discussion

We recently investigated the microbiome associated with OLK, a potentially malignant lesion with the potential to transform to OSCC. Unexpectedly, these studies identified increased levels of *R. mucilaginosa* on OLK. This was surprising as previous studies of OSCC have shown that the abundance *R. mucilaginosa* is decreased at oral cancers [1,38]. Although *R. mucilaginosa* is generally absent or reduced at OSCCs, our data do not preclude the possibility that the increased abundance of this organism on OLK could play a role in the malignant transformation to OSCC through the production of toxic metabolites such as ACH. The predictive metagenome (PICRUSt) analysis presented here showed that bacterial communities found on OLK have significantly fewer genes encoding ALDH compared to healthy mucosa. This reduced expression of ALDH enzymes, which are required to metabolise ACH, would reduce the capacity of these OLK communities to remove toxic and carcinogenic ACH from the vicinity of the oral mucosa. Our analysis of oral bacterial genomes indicate that this change in the OLK metagenome is likely due to the decreased abundance of streptococci (which generally encode ALDH) and the increased abundance of *R. mucilaginosa* (which generally do not encode ALDH) on OLK. Our analysis of the 70 genomes corresponding to *Rothia* spp. in the PATRIC database showed that only two genes encoding ALDHs were present in this group of strains. The genes were present in two strains of *R. mucilaginosa* recovered from bronchoalveolar lavage specimens from intensive care patients [39] and the genes present are likely the result of recent horizontal gene transfer events. None of the oral isolates examined encoded ALDH. Interestingly, a high proportion (69%) of strains of *Neisseria* spp. harboured a predicted ALDH encoding gene. Previous studies have shown that oral strains of *Neisseria* lack enzymatic ALDH activity and generate high levels of ACH, demonstrating that in *Neisseria* spp., possession of an ALDH encoding gene does not necessarily translate to high levels of enzymatic ALDH activity [18].

Next, we decided to isolate *R mucilaginosa* from OLK patients to determine their capacity to generate ACH from ethanol. We initially used a colorimetric assay which detects total aldehyde production. Under the assay conditions, this method produced very similar results to GC-MS analysis for acetaldehyde using representative strains (Figure 3(b)). Previous studies with oral streptococci and *Neisseria* spp. have shown that bacteria that lack ALDH activity have increased capacity to generate ACH in the presence of ethanol. Our analysis of 8 *R. mucilaginosa* strains here shows that, in the presence of 100 mM ethanol, most of the isolates (6/8) produced acetaldehyde in the range of 40–60 µM, with one isolate (41C2) generating on average >150 µM ACH detected by GC-MS. These ACH levels were similar to, or in the case of isolate 41C2, in excess of those detected with isolates of *N. mucosa* and *C. albicans*, microbes previously implicated as the primary sources of microbial ACH in the oral cavity. All of these *R. mucilaginosa* isolates were recovered from patients with a history of alcohol consumption placing them at risk of ACH exposure (Table S1). The absence of an ALDH encoding gene has also been associated with increased susceptibility to ethanol in bacteria [15]. All bacteria here were inhibited in the presence of 4% (~0.87 M) ethanol. The ALDH positive streptococci were unaffected by the presence of 2% (~0.44 M) ethanol whereas the acetaldehyde producers (*Rothia, Neisseria*) exhibited increased susceptibility to growth inhibition by 2% ethanol.

These studies demonstrate that *R. mucilaginosa*, an important component of the normal oral microbiome can produce ACH. Recently, Yokoyama *et al*. (2018) showed that individuals with the highest capacity to generate ACH in saliva had increased levels of *R. mucilaginosa* and *S. salivarius* in their salivary microbiome [24]. Our data would support a direct role for *R. mucilaginosa* in generating ACH in those individuals. Our previous studies of the microbiome of OLK identified higher levels of *R. mucilaginosa* on OLK relative to healthy NC tissue from the same patients. This species shift reduces ALDH functionality in the metagenome which could lead to local build-up of ACH at the site of the OLK. If this hypothesis is true, local ACH production may contribute to the malignant transformation of these lesions. Alternatively, the species shift may have predated the OLK and contributed to the development of the OLK itself. This study indicates that the levels of ACH produced by *R. mucilaginosa* are capable of inducing oxidative stress in oral keratinocytes, indicating that this level of ACH production is biologically significant. The ACH levels detected in these experiments are similar to the concentrations that can be detected in the oral cavity after alcohol consumption [13].

In conclusion, our earlier work has demonstrated increased prevalence of *R. mucilaginosa* in patients at OLK sites compared to at contralateral normal sites. This current study provides further evidence to implicate *R. mucilaginosa* in the pathogenesis of OLK. Its presence is likely to lead to increased ACH production in the oral cavity which could be involved in the development of OLK *ab initio* and/or the malignant transformation of these lesions, especially in those patients who drink alcohol. *R. mucilaginosa* requires further investigation as a contributor to the oral ACH burden following alcohol consumption. If this can be confirmed, strategies to control *R. mucilaginosa* could be useful in preventing the development of OLK in those who drink alcohol and/or reducing the malignant transformation of OLK to OSCC.

## Supplementary Material

Supplemental Material
