# Supplementary material for: Acetaldehyde production by Rothia mucilaginosa isolates from patients with oral leukoplakia
Source: J Oral Microbiol. 2020 Mar 21;12(1):1743066. doi: 10.1080/20002297.2020.1743066 (PMC7170386; doi:10.1080/20002297.2020.1743066)
Supplement: Supplemental Material [file ZJOM_A_1743066_SM4276.docx]

**Table S1.** *R. mucilaginosa* isolates included in this study, indicating the patient type and whether the patient was a smoker or alcohol consumer.

| Patient Num. | Sample ID | Patient* | Smoker | Alcohol consumption |
| --- | --- | --- | --- | --- |
| NA | *R. mucilaginosa* DY-18 (DSM-20746) | AP | N/A | N/A |
| 1 | *R. mucilaginosa* 40A1 | OLK | yes | yes |
| 2 | *R. mucilaginosa* 41C2 | OLK | yes | yes |
| 3 | *R. mucilaginosa* 43B1 | OLK | yes | yes |
| 4 | *R. mucilaginosa* 44A1 | OLK | no | yes |
| 5 | *R. mucilaginosa* 107A | OLK | yes | yes |
| 6 | *R. mucilaginosa* 66A9 | OLK | yes | yes |
| 7 | *R. mucilaginosa* H2.2 | HC | no | yes |
| 8 | *R. mucilaginosa* H4.2 | HC | no | no |

*AP: Apical Periodontitis; OLK: oral leukoplakia; NC: Contralateral normal; HC: healthy control.

**Table S2.** Doubling times (h) of indicated isolates in the absence and presence of ethanol in BHI broth at 37˚C. NG=No Growth.

| Strains | 0.0% Ethanol | 2% Ethanol | 4% Ethanol |
| --- | --- | --- | --- |
|  | Doubling time (95% CI) | Doubling time (95% CI) | Doubling time (95% CI) |
| *S. mitis* NCTC12261 | 0.9886 (0.8385 to 1.165) | 0.9789 (0.8442 to 1.135) | NG |
| *S. gordonii* DL1 | 1.015 (0.7925 to 1.297) | 0.8598 (0.7989 to 0.9247) | 1.747 (1.539 to 1.999) |
| *C. albicans* 132A | 1.333 (0.6782 to 3.037) | 1.004 (0.7197 to 1.403) | 0.7119 (0.5345 to 0.9277) |
| *N. mucosa* DSM17611 | 2.223 (1.763 to 2.842) | 2.401 (1.886 to 3.113) | NG |
| *R. mucilaginosa* DY18 | 0.7409 (0.8995 to 1.264) | 1.111 (1.17 to 2.251) | NG |
| *R. mucilaginosa* 41C2 | 0.5864 (0.4299 to 0.7737) | 0.6118 (0.4694 to 0.78) | 1.017 (0.3052 to 3.716) |
